# Developing an evaluation indicators of health literacy for cervical cancer among Chinese women: a modified Delphi method study

**DOI:** 10.1186/s12885-023-11208-3

**Published:** 2023-09-12

**Authors:** Chanchan He, Chenyang Pei, Jing Ma

**Affiliations:** 1 Institute for Hospital Management of Tsinghua University, Shenzhen, 518055 China; 2 School of Health Policy Management, Peking Union Medical College, Beijing, 100730 China

**Keywords:** Cervical cancer, Health literacy, Evaluation index system, Delphi, China

## Abstract

**Background:**

Increasing women’s health literacy is the key to preventing cervical cancer, and various tools have been developed to assess women’s cancer health literacy. However, many of these tools come from other countries and have not been adapted to Chinese requirements. Furthermore, a system for evaluating cervical cancer health literacy among Chinese women has not been developed. Therefore, we sought to establish an evaluation index system for cervical cancer health literacy among Chinese women and to provide an effective evaluation tool for tertiary prevention of cervical cancer in China.

**Methods:**

We invited 20 recognized experts to participate in two rounds of Delphi expert consultation, and the modified Delphi process with percentage weighting and multiplication was used. A literature review identified 67 potential indicators. Subsequent discussions within our research team led to the retention of 48 indicators following a rigorous screening process. On this basis, two rounds of Delphi expert consultation were conducted to rate and screen the indexes. Percentage weighting and multiplication were used to determine index weights.

**Results:**

Twenty experts participated in the first-round Delphi consultations (95.23% recovery rate). In the second-round Delphi consultations, 20 questionnaires were returned (100%), and the expert authority coefficient was 0.93 ± 0.02. After both rounds of Delphi consultation, 4 first-level indicators, 9 second-level indicators, and 32 third-level indicators were identified for cervical cancer literacy among Chinese women. On a five-point scale, importance ratings ranged from 3.76 to 4.95 points, with variation coefficients ranging from 0.06 to 0.25, while sensitivity ratings ranged from 3.71 to 4.83 points, with variation coefficients ranging from 0.08 to 0.24. Across both rounds, Kendall’s *W* coefficients ranged from 0.168 to 0.248. The weights of first-level indicators of basic knowledge and attitudes about cervical cancer, primary prevention of cervical cancer literacy, secondary prevention of cervical cancer literacy, and tertiary prevention of cervical cancer literacy were 0.257, 0.249, 0.251, and 0.243, respectively.

**Conclusions:**

We have developed the first tertiary prevention-based, comprehensive evaluation index system for cervical cancer literacy among Chinese women, which will provide theoretical support for cervical cancer prevention and health education programs.

**Supplementary Information:**

The online version contains supplementary material available at 10.1186/s12885-023-11208-3.

## Introduction

Cervical cancer is the fourth-most prevalent cancer among women worldwide. It is a significant public health issue, resulting in approximately 604,000 new cases and 342,000 deaths in 2020 [1].Moreover, about 90% of cervical cancer cases and deaths occur in low- and middle-income countries [2]. In 2016, China reported 32,000 cervical cancer cases and 10,000 deaths, ranking it the eighth leading cause of cancer-related deaths in women[3].Over the past decade, China has experienced an increase in cervical cancer incidence from 5.4 to 12.3 per 100,000 and mortality rates from 1.1 to 3.5 per 100,000, contributing to a growing disease burden [4].

Human papillomavirus (HPV) infection causes 90% percent of cervical cancers. Thus, the adoption of primary and secondary prevention methods can effectively prevent cervical cancer [5, 6]. The World Health Assembly adopted a global strategy in August 2020 to eliminate cervical cancer. This strategy set a goal for all countries to achieve and maintain an incidence rate of less than four per 100,000 women. Additionally, the plan proposed adopting a tertiary prevention strategy by 2030 to eliminate cervical cancer in the future [7]. Notably, developed countries have witnessed a decline in cervical cancer incidence due to the implementation of effective cervical screenings and the provision of free HPV vaccinations [8–10]. Cervical cancer screening and free HPV vaccination have reduced cervical cancer mortality rates. However, the success of these programs relied heavily on public awareness of cervical cancer prevention [11].

The concept of cervical cancer health literacy refers to individuals’ capacity to obtain, process, and comprehend basic health information and services that will enable them to make appropriate health decisions. Improving cervical cancer health literacy levels is one of the keys to preventing cervical cancer [12]. Several studies have shown that an increased level of cervical cancer health literacy is associated with improved HPV vaccination, cervical cancer screening, and treatment [13–15]. In China, the HPV vaccine is not part of the national immunization program. However, a cervical cancer screening program was initiated in 2009 for rural women aged 35–64 as part of primary healthcare. Despite this, a recent study found that only 25.7% of women aged 20–64 in China had undergone previous screening for cervical cancer in 2015 [16]. Based on the number of HPV vaccine doses administered nationwide, it is estimated that, in 2020, the HPV vaccination rate for women aged 9–45 years was 2.24% in China [17]. However, rates of HPV vaccine uptake and cervical cancer screening in China are low, indicating a lack of health literacy. Despite the crucial role of health literacy in cervical cancer prevention, adult women in many regions are estimated to have limited or basic cervical cancer health literacy levels [18–22]. A valid and reliable instrument for assessing cervical cancer health literacy is the first step in developing interventions to increase knowledge and uptake of cervical cancer prevention services. In China, most cervical cancer health literacy surveys use self-developed questionnaires that have not been developed following a comprehensive, integrated, and systematic process [23–25]. Furthermore, foreign evaluation tools are also not suitable for Chinese women [26]. Therefore, this study sought to create an evaluation indicator for cervical cancer health literacy in order to assess women’s knowledge about the tertiary prevention of cervical cancer and to provide a more scientific basis for the development of assessment tools.

## Methods

### Establishing a preliminary pool of indicators

To identify potential cervical cancer health literacy indicators, a comprehensive literature search was conducted in various databases, including PubMed, Web of Science, China National Knowledge Infrastructure (CNKI), China Science and Technology Journal Database, and Wanfang Data. The search covered articles published from January 1, 2020, to December 31, 2021. The retrieval strategy employed the following: ((((((cervical cancer [Title]) *(cervical intraepithelial neoplasia [Title])) *(HPV vaccine [Title])) *(HPV [Title])) *(human papillomavirus [Title])) *(cervical cancer screening [Title])) and (((knowledge [Title]*(health literacy [Title]))) + (((cervical cancer [Title])) *(cervical intraepithelial neoplasia [Title]))) and (tertiary prevention [[Title/Abstract]). After assessing the abstracts and eliminating irrelevant articles, 270 references were considered for further analysis (see Additional file 1). The following four major knowledge domains were identified as a result of a comprehensive literature review: (1) basic knowledge and attitudes about cervical cancer, (2) knowledge of cervical cancer primary prevention measures, (3) knowledge of cervical cancer secondary prevention measures, and (4) knowledge of cervical cancer tertiary prevention measures. Our study also included indicators from a variety of validated tools that cover a broader spectrum of cervical cancer knowledge assessment [19, 27–29].

**Fig. 1:**
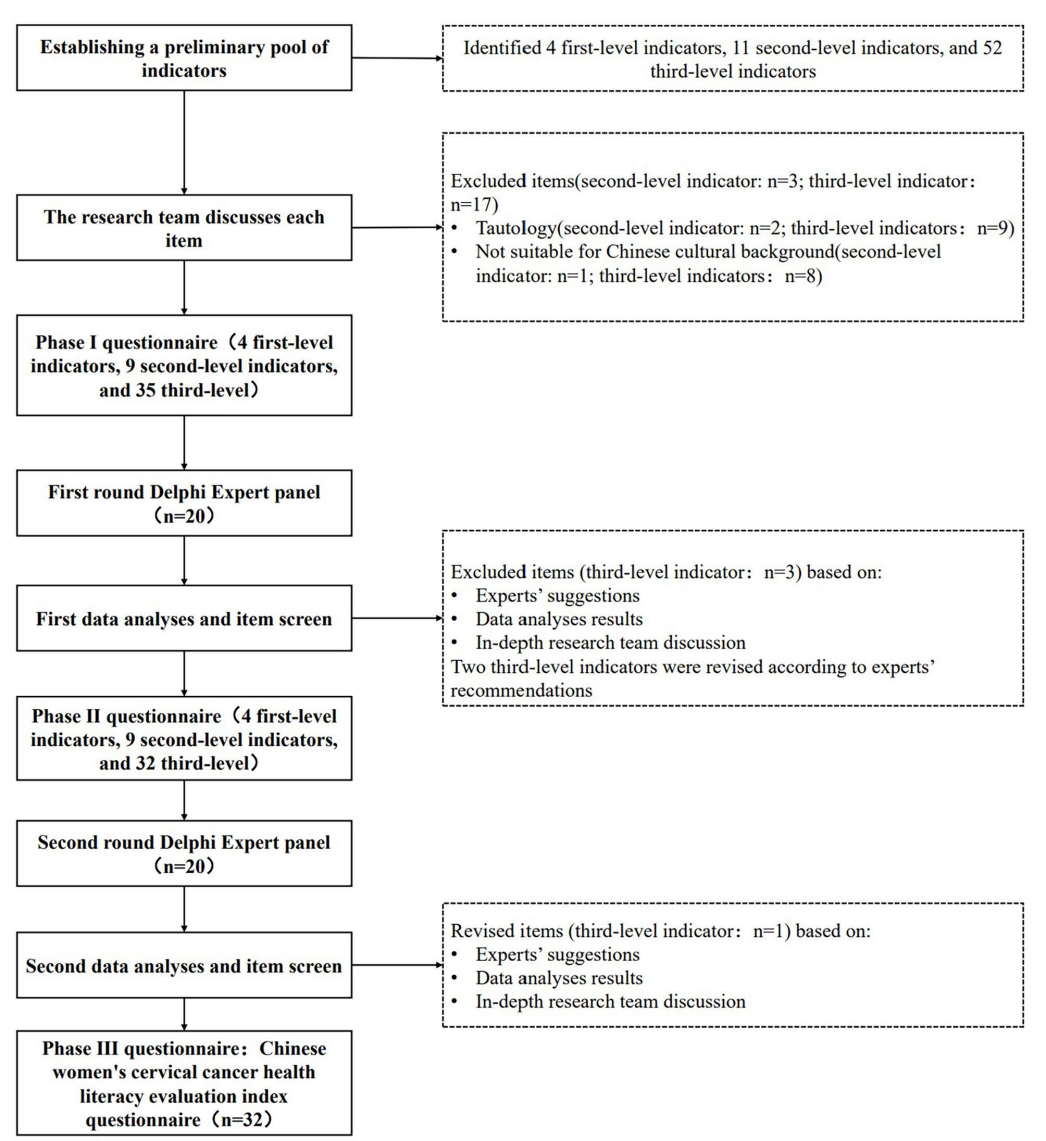
Flow chart of items selection of Chinese women’s cervical cancer health literacy evaluation indicators

We then conducted an expert consultation to refine the potential quality indicator pool. Individuals from the fields of health education and health promotion, doctors who treat cervical cancer patients, and women’s health researchers discussed whether the indicators were suitable and valid. As part of the modified Delphi consultation protocol, none of these experts participated. Using these two steps, the modified Delphi consultation included 4 first-level indicators, 9 second-level indicators, and 32 third-level indicators (see Additional file 2). The complete flow chart for item selection in the Chinese women’s cervical cancer health literacy evaluation indicators is presented in Fig. 1.

### Development of an index system using the Delphi method

#### Data collection

On the basis of the preliminary indicator pool, a Delphi consultation questionnaire was designed to collect expert opinions. The questionnaire consisted of the following three sections: (1) information about the experts, such as their gender, age, and years of experience; (2) content of the Delphi expert consultation, where experts rated each indicator in terms of importance and sensitivity using a Likert 5-level scoring method with 1–5 points indicating answers ranging from “very unimportant/sensitive” to “very important/sensitive”; and (3) familiarity scale with cervical cancer health literacy on a scale of 1 (very unfamiliar) to 5 (very familiar) points. Additionally, experts were consulted about each indicator’s deletions and additions and their remarks were included in a comments column.

#### Selection by experts

To meet the minimum requirement of 15 experts as stipulated by the Delphi expert consultation method, we initially planned to select a panel of 15–20 experts based on specific circumstances. To ensure the expertise and credibility of the experts and to consider the regional disparities in cervical cancer prevention and control, we randomly invited experts from provincial-level maternal and child health hospitals, disease control centers, health bureaus, and health education centers across China. Ultimately, we selected a diverse group of twenty experts from various regions, encompassing specialties such as women’s health, cervical cancer diagnosis and treatment research, health education, and disease prevention. Experts who participated in the Delphi consultations were required to (1) have worked in their respective field for more than 10 years, (2) be familiar with the research topic, and (3) be able to provide comprehensive opinions and participate enthusiastically in both rounds of consultation.

#### Delphi procedure

In June and September 2022, Delphi expert consultations were conducted via email. In the first round, experts ranked each potential indicator’s importance and sensitivity on a five-point Likert scale. To ensure that the experts understood each indicator, they were defined in detail. Experts could also make comments about each indicator or suggest adding or removing specific indicators. Using indicator screening criteria, we collected open opinions on the indicators in the first round, deleted and revised them, and sent them for the second round of expert consultations. Experts’ scores on the importance and sensitivity of each indicator were collected again during the second round of expert consultations, and the final indicator system was created. Similarly, the same 20 experts were invited to complete the questionnaires in the second round.

Screening criteria included an average score of > 3.50 points for indicator importance and sensitivity and a coefficient of variation of < 0.25 as the quantitative standard for indicator retention. If the indicators did not meet the above criteria or if the experts recommended adding, modifying, or deleting them, the research team made a final decision after discussing and considering all suggestions [30].

#### Determining the subjective weights

After the Delphi consultation, the percentage weighting method was used to calculate the weight of the primary indicators, and the percentage weight method combined with the production method was used to calculate the weights and combined weights of the secondary and tertiary indicators. First, the importance of each indicator was rated (1–5 points) by experts during the second round of consultations, and the average value was calculated. To determine the weight of the primary indicator, the average score of that indicator was divided by the sum of all the primary indicators’ average scores. A secondary index’s percentage weight was calculated by multiplying its percentage weight by its weight in the primary index it belonged to, using the same primary index as a whole. As a result, the combined weight of the secondary index was obtained. Finally, we calculated the weight and combined weight of the tertiary index [31].

### Analysis and management of data

SPSS version 26.0 (IBM Corporation, Armonk, NY, USA) and Excel 2010 (Microsoft Corporation, Redmond, WA, USA) were used to calculate the mean, standard deviation, and coefficient of variation values of each index’s importance and sensitivity scores, as well as the expert’s positive coefficient, expert’s authority coefficient, and weights of indicators at all levels. SPSS version 26.0 was used to calculate the Kendall *W* coefficient of concordance of the experts’ evaluation of the importance and sensitivity of the indicators during both rounds of consultations.

## Results

### Characteristics of the Delphi participants

The first-round Delphi consultation involved 20 experts (the recovery rate was 95.23%). For the second round of Delphi consultation, revised questionnaires were sent to the same 20 experts, and a total of 20 questionnaires were returned (for a recovery rate of 100.00%). As shown in Table 1, the 20 experts had different characteristics. Almost all included experts had high academic achievements, with 16 (100%) holding senior associate titles or higher and 20 (100.00%) working in relevant fields for more than 10 years. The average authoritative coefficient was calculated to be 0.93 ± 0.02.

**Table 1:** Characteristics of experts (*N* = 20)

Characteristic		n	Percentage (%)
Sex			
	Male	3	15.00
	Female	17	85.00
Age(years)			
	< 40	2	10.00
	41–50	6	30.00
	51–60	10	50.00
	> 60	2	10.00
Work experience(years)			
	< 10	0	0.00
	10–19	4	20.00
	20–29	5	25.00
	≥ 30	11	55.00
Profession title			
	Senior	12	60.00
	Senior deputy	8	40.00
Education			
	Associate	1	5.00
	Undergraduate	9	45.00
	Master’s	6	38.00
	PhD	4	20.00
Area of expertise (multiple choice)			
	Women's health	8	33.33
	Cervical cancer diagnosis and treatment	6	25.00
	Health education and health promotion	5	20.83
	Disease prevention and control	5	20.83

### Concentration and variation of expert opinions

An additional 1 shows the concentration and variation of expert opinions. According to the first round, the mean importance scores for potential indicators ranged from 3.79 to 4.89 points, with variation coefficients ranging from 0.06 to 0.25, while the mean sensitivity scores ranged between 3.74 and 4.68 points, with variation coefficients ranging from 0.11 to 0.28. Following the second round, potential indicators scored between 3.76 and 4.95 points on the importance scale, with variation coefficients varying from 0.04 to 0.24, while the potential indicators’ sensitivity scores ranged between 3.71 and 4.83, with variation coefficients ranging from 0.08 to 0.24.

### Coordination of experts’ opinions

The coordination of experts’ opinions is shown in Table 2. Kendall’s *W* coefficients ranged between 0.168 and 0.248 in both rounds, and the importance and sensitivity scores in both rounds were all effective (*p* < 0.01), suggesting consistency among experts.

**Table 2:** Degree of coordination among expert opinions

	First round		Second round	
	Importance	Sensitivity	Importance	Sensitivity
*W*	0.203	0.168	0.223	0.248
*χ* ^*2*^	150.314	123.954	144.763	154.06
*P*	< 0.0001	< 0.0001	< 0.0001	< 0.0001

Note: The number of indicators in the first round, which included 20 experts, was 39; the number of indicators in the second round, which also included 20 experts, was 32

### Modification of indicators

In the first round of the study, three third-level indicators (awareness of the policy on free HPV vaccination, whether HPV infection is treatable, and persistent pelvic pain) were deleted, and two third-level indicators were revised (abnormal vaginal bleeding and abnormal vaginal discharge). As a result, 4 first-level, 9 second-level, and 32 third-level indicators were included in the second round of consultation. Then, as part of the second round of revisions, a third-level indicator was revised (significance of abnormal cervical cancer screening results). Ultimately, there were 4 first-level indicators, 9 second-level indicators, and 32 third-level indicators (see Additional file 2).

### Indicator weight results

According to the importance of each indicator during the second round of expert correspondence, we calculated the weights of the primary indicators using the percentage-weighting method and the weights of the secondary and tertiary indicators using the percentage-weighting and multiplication methods. Table 3 shows the results.

**Table 3:** Final index system

Primary indicators	Weight	Secondary indicators	Combined weight	Tertiary indicators	Combined weight
1. Basic knowledge and attitudes about cervical cancer	0.257	1.1 Basic knowledge	0.089	1.1.1 Epidemiologic characteristics of cervical cancer	0.029
				1.1.2 Early-stage cervical cancer is preventable	0.031
				1.1.3 Early-stage cervical cancer is curable	0.030
		1.2 Basic attitudes	0.087	1.2.1 Perceived severity of cervical cancer	0.087
		1.3 Policy knowledge	0.081	1.3.1 Awareness rate of screening program policy	0.081
2. Cervical cancer primary prevention literacy	0.249	2.1 Risk factors for cervical cancer	0.085	2.1.1 HPV infection	0.010
				2.1.2 Long-term smoking	0.009
				2.1.3 Long-term use of oral contraceptives (birth control pills or estrogens)	0.008
				2.1.4 Becoming sexually active at a young age (especially < 18 years old)	0.010
				2.1.5 Young age at first full-term pregnancy or having multiple full-term pregnancies	0.009
				2.1.6 Having a family history of cervical cancer	0.009
				2.1.7 Suffering from genital infections and other sexually transmitted diseases (*Chlamydia* infection)	0.010
				2.1.8 Having many sexual partners or having one partner who is considered high risk (someone with HPV infection or who has many sexual partners)	0.010
				2.1.9 Having a weakened immune system (HIV infections or taking drugs to suppress their immune response)	0.009
		2.2 Basic knowledge of HPV	0.082	2.2.1 HPV susceptibility in young women	0.020
				2.2.2 Signs and symptoms of HPV infection	0.020
				2.2.3 Infection by the HPV is the most important risk factor for cervical cancer	0.022
				2.2.4 Ways to prevent HPV	0.020
		2.3 HPV vaccination	0.082	2.3.1 HPV vaccine can effectively protect against 70–90% of cervical cancers	0.021
				2.3.2 Optimal age range for HPV vaccination	0.021
				2.3.3 Women need regular cervical screening even after HPV vaccination	0.021
				2.3.4 Attitudes and intentions toward HPV vaccination	0.019
3. Cervical cancer secondary prevention literacy	0.251	3.1 Signs and symptoms of cervical cancer	0.122	3.1.1 Abnormal vaginal bleeding (when not on your period or after periods have stopped or bleeding after intercourse)	0.062
				3.1.2 Abnormal vaginal discharge (which appears pale, brown, pink, watery, or contains blood)	0.060
		3.2 Cervical cancer screening	0.129	3.2.1 Common cervical cancer screening methods	0.019
				3.2.3 The most appropriate age to start screening women for cervical cancer	0.018
				3.2.4 The frequency of cervical cancer screening	0.019
				3.2.5 Next steps after an abnormal cervical cancer screening test	0.018
				3.2.6 Things you need to know before your first cervical screening	0.018
				3.2.7 Cervical cancer screening experience in the past 3 years	0.018
				3.2.8 Perceived benefits of cervical cancer screening	0.019
4. Cervical cancer tertiary prevention literacy	0.243	4.1 Seek medical attention on time	0.243	4.1.1 Attitude toward timely access to medical care	0.243

Abbreviations: HIV, human immunodeficiency virus; HPV, human papillomavirus

## Discussion

Based on both rounds of modified Delphi consultation, we developed a system for evaluating Chinese women’s cervical cancer health literacy and determined the weights of all indicators. There are 4 first-level indicators, 9 second-level indicators, and 32 third-level indicators in this system, which provides a scientific reference for improving cervical cancer health literacy among Chinese women.

In terms of research methods, the response rates in both rounds of Delphi consultation were high, indicating that the experts were committed to this project and appreciated its significance. The study’s experts were all highly educated and had extensive work experience in diverse fields, including women’s health, cervical cancer diagnosis and treatment research, health education, and disease prevention. There was a robust result, as the authoritative coefficient was 0.93. The Kendall’s W test scores ranged between 0.168 and 0.248 in both rounds, indicating that the results of the indicator system are reliable and can describe and explain the level of cervical cancer health literacy among Chinese women scientifically, accurately, and reliably.

In terms of indicator content, the indicator system includes basic knowledge and attitudes about primary, secondary, and tertiary prevention measures of cervical cancer, and it focuses on all aspects of tertiary prevention of cervical cancer and can serve as a reference for cervical cancer prevention efforts, enabling a more comprehensive assessment of cervical cancer health literacy. In other countries, cervical cancer health literacy scales have been developed, but these scales primarily focus on perceptions of cervical cancer’s severity and susceptibility as well as health behavior [20, 27, 32]. As a result, they do not provide a comprehensive view of the cervical cancer tertiary prevention process as a whole. In some studies, both primary and secondary prevention of cervical cancer were examined; one used a questionnaire for measuring knowledge about cervical cancer among women in Oman aged 20–65 years that contained four main domains of knowledge, including knowledge about general cervical cancer, knowledge about risk factors associated with the disease, and knowledge about primary and secondary prevention [33]. In contrast to the evaluation system we developed for cervical cancer health literacy among Chinese women, this earlier questionnaire did not include information on tertiary prevention of cervical cancer, such as seeking medical attention on time to treat early cervical cancer. Several assessments of cervical cancer knowledge and prevention are also available, but they focus primarily on female high school and university students [34, 35].

Among the four first-level indicators in this study, the weights of basic knowledge and attitudes about cervical cancer, cervical cancer primary prevention literacy, cervical cancer secondary prevention literacy, and cervical cancer tertiary prevention literacy were 0.257, 0.249, 0.251, and 0.243, respectively. According to the weighted indicators, cervical cancer secondary prevention literacy (0.251) received the highest score, which indicates that women need to be educated on cervical cancer screening, early detection, and the symptoms of cervical cancer. Meanwhile, cervical cancer tertiary prevention literacy received the lowest weighted indicator (0.243), which indicates that experts believe that knowledge regarding seeking timely medical care after a cervical cancer diagnosis is relatively less important for the average woman. This is in line with the needs of health education work and suggests that cervical cancer patients could be the focus of this part of the work.

The assessment indicators prioritized tertiary prevention to reduce cervical cancer incidence and mortality in Chinese women. It identified knowledge gaps in cervical cancer prevention and provided targeted educational materials and interventions to increase awareness of cervical cancer and its risk factors. This evaluation indicator can give a more comprehensive assessment of cervical cancer health knowledge among Chinese women. It can help policymakers assess the effectiveness of prevention efforts, identify areas of low ability, and inform targeted policies and interventions. Collaboration between researchers, healthcare providers, and policymakers is crucial for effectively implementing the evaluation system in China’s cervical cancer prevention and health education programs. We suggested integrating the system with existing prevention efforts, using culturally appropriate indicators, and continuously evaluating program effectiveness. This study developed an indicator system suitable for Chinese women for cervical cancer health literacy by fully incorporating existing knowledge indicators for tertiary prevention of cervical cancer. We conducted an extensive literature review and expert interviews to ensure comprehensiveness and representativeness. The inclusion of experts in various fields of cervical cancer prevention assured the authority and validity of the Delphi survey data. However, as Chinese women lack knowledge about cervical cancer prevention, we did not interview them for cervical cancer health knowledge indicators. Instead, experts were asked to select indicators from the perspective of the average Chinese woman. Although the indicator system has not yet been implemented in a large sample of Chinese women, we plan to validate its reliability and validity in a follow-up study.

## Conclusion

In our study, following the Delphi method strictly, we reached a good consensus on 45 indicators and determined the weights of indicators at all levels using percentage weights and multiplication. This is also the first study to develop a tertiary prevention-based, comprehensive evaluation index system for cervical cancer literacy among Chinese women, which will provide theoretical support for cervical cancer prevention and health education programs.

### Electronic supplementary material

Below is the link to the electronic supplementary material.

Additional file 1

Additional file 2

## Data Availability

All data generated or analyzed during the study have been included in the published article (and its additional files).
