# Supplementary material for: Developing an evaluation indicators of health literacy for cervical cancer among Chinese women: a modified Delphi method study
Source: BMC Cancer. 2023 Sep 12;23:863. doi: 10.1186/s12885-023-11208-3 (PMC10498636; doi:10.1186/s12885-023-11208-3)
Supplement: Supplementary file 1 — Additional file 1 [file 12885_2023_11208_MOESM1_ESM.doc]

**Additional file 1**

**Databases:**

1. PubMed: <https://pubmed.ncbi.nlm.nih.gov/?db=PubMed>
2. Web of Science: <https://www.webofscience.com/wos/woscc/basic-search>
3. China National Knowledge Infrastructure (CNKI): https://www.cnki.net/
4. Wanfang Data: <https://www.wanfangdata.com.cn/>
5. China Science and Technology Journal Database: <http://www.cqvip.com/>

**Search strategy:**

((((((cervical cancer [Title]) *(cervical intraepithelial neoplasia [Title])) *(HPV vaccine [Title])) *(HPV [Title])) *(human papillomavirus [Title])) *(cervical cancer screening [Title])) and (((knowledge [Title]*(health literacy [Title]))) + (((cervical cancer [Title]) *(cervical intraepithelial neoplasia [Title]))) and (tertiary prevention [Title/Abstract])

**Date limitations:**

Published from January 1, 2020, to December 31, 2021.

**Language limitations:**

1. English
2. Chinese


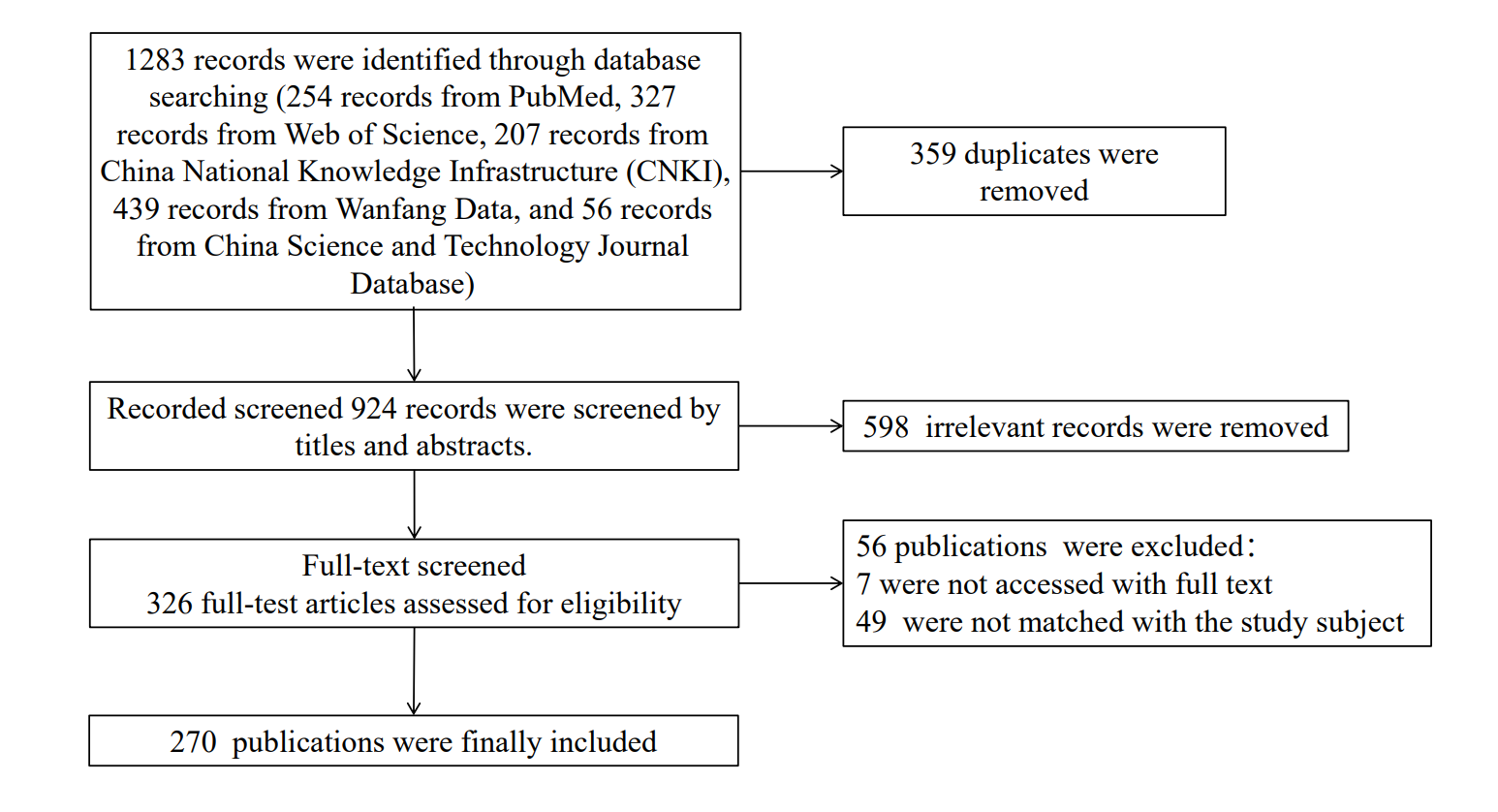


Figure S1. Flow chart for selection of the studies
