# Supplementary material for: Developing an evaluation indicators of health literacy for cervical cancer among Chinese women: a modified Delphi method study
Source: BMC Cancer. 2023 Sep 12;23:863. doi: 10.1186/s12885-023-11208-3 (PMC10498636; doi:10.1186/s12885-023-11208-3)
Supplement: Supplementary file 2 — Additional file 2 [file 12885_2023_11208_MOESM2_ESM.doc]

**Additional file 2**

Table S1. Results of two rounds of expert consultation on the proposed indicators

| **Indicator** | **Round 1** | | | | | **Round 2** | | | | |
| --- | --- | --- | --- | --- | --- | --- | --- | --- | --- | --- |
|  | **Significance** | | **Sensitivity** | | **Outcome** | **Significance** | | **Sensitivity** | | **Outcome** |
|  | **X ± SD** | **CV** | **X ± SD** | **CV** |  | **X ± SD** | **CV** | **X ± SD** | **CV** |  |
| 1. **Basic knowledge and attitudes about cervical cancer** | 4.68 ± 0.46 | 9.92 | 4.37 ± 0.74 | 16.95 | Accepted | 4.79 ± 0.40 | 8.31 | 4.45 ± 0.59 | 13.25 | Accepted |
| **1.1 Basic knowledge** | 4.42 ± 0.82 | 18.44 | 4.53 ± 0.82 | 18.09 | Accepted | 4.71 ± 0.44 | 9.45 | 4.61 ± 0.48 | 10.40 | Accepted |
| 1.1.1 Epidemiologic characteristics of cervical cancer | 4.11 ± 0.85 | 20.75 | 4.05 ± 0.83 | 20.37 | Accepted | 4.02 ± 0.71 | 17.69 | 4.01 ± 0.63 | 15.80 | Accepted |
| 1.1.2 Early-stage cervical cancer is preventable | 4.89 ± 0.31 | 6.27 | 4.58 ± 0.49 | 10.78 | Accepted | 4.95 ± 0.22 | 4.41 | 4.78 ± 0.51 | 10.72 | Accepted |
| 1.1.3 Early-stage cervical cancer is curable | 4.79 ± 0.52 | 10.88 | 4.37 ± 0.74 | 16.95 | Accepted | 4.78 ± 0.40 | 8.36 | 4.62 ± 0.65 | 14.15 | Accepted |
| **1.2 Basic attitudes** | 4.53 ± 0.50 | 11.03 | 4.47 ± 0.82 | 18.30 | Accepted | 4.57 ± 0.49 | 10.63 | 4.16 ± 0.57 | 13.75 | Accepted |
| 1.2.1 Perceived severity of cervical cancer | 4.32 ± 0.73 | 16.90 | 4.26 ± 0.71 | 16.75 | Accepted | 4.48 ± 0.58 | 12.97 | 4.22 ± 0.75 | 17.73 | Accepted |
| **1.3 Policy knowledge** | 4.26 ± 0.96 | 22.63 | 4.26 ± 0.96 | 22.63 | Accepted | 4.26 ± 0.77 | 17.98 | 3.95 ± 0.80 | 20.37 | Accepted |
| 1.3.1 Awareness of screening program policy | 4.26 ± 0.85 | 19.91 | 4.11 ± 0.91 | 22.21 | Accepted | 4.01 ± 0.71 | 17.66 | 3.75 ± 0.62 | 16.60 | Accepted |
| 1.3.2 Awareness of the free HPV vaccination program | 3.79 ± 0.95 | 25.08 | 3.74 ± 0.96 | 25.82 | Deleted |  | | | | |
| 1. **Cervical cancer primary prevention literacy** | 4.89 ± 0.31 | 6.27 | 4.37 ± 0.74 | 16.95 | Accepted | 4.64 ± 0.57 | 12.24 | 4.52 ± 0.49 | 10.83 | Accepted |
| **2.1 Risk factors for cervical cancer** | 4.68 ± 0.57 | 12.10 | 4.58 ± 0.67 | 14.72 | Accepted | 4.63 ± 0.57 | 12.27 | 4.21 ± 0.75 | 17.74 | Accepted |
| 2.1.1 HPV infection | 4.58 ± 0.67 | 14.72 | 4.47 ± 0.68 | 15.16 | Accepted | 4.68 ± 0.45 | 9.73 | 4.46 ± 0.58 | 13.08 | Accepted |
| 2.1.2 Long-term smoking | 4.32 ± 0.57 | 13.13 | 4.16 ± 0.67 | 16.11 | Accepted | 4.26 ± 0.70 | 16.34 | 4.05 ± 0.67 | 16.52 | Accepted |
| 2.1.3 Long-term use of oral contraceptives (birth control pills or estrogens) | 4.21 ± 0.61 | 14.58 | 4.21 ± 0.69 | 16.49 | Accepted | 3.76 ± 0.89 | 23.68 | 3.71 ± 0.90 | 24.34 | Accepted |
| 2.1.4 Becoming sexually active at a young age (especially <18 years old) | 4.63 ± 0.58 | 12.55 | 4.37 ± 0.74 | 16.95 | Accepted | 4.32 ± 0.84 | 19.44 | 4.11 ± 0.83 | 20.21 | Accepted |
| 2.1.5 Young age at first full-term pregnancy or having multiple full-term pregnancies | 4.26 ± 0.64 | 14.92 | 4.11 ± 0.72 | 17.48 | Accepted | 4.22 ± 0.75 | 17.73 | 3.96 ± 0.67 | 16.93 | Accepted |
| 2.1.7 Having a weakened immune system (HIV infections or taking drugs to suppress immune response) | 4.05 ± 1.00 | 24.67 | 4.04 ± 0.88 | 21.70 | Accepted | 4.06 ± 0.87 | 21.31 | 4.01 ± 0.84 | 20.90 | Accepted |
| 2.1.8 Having a family history of cervical cancer | 4.32 ± 0.65 | 15.13 | 4.37 ± 0.67 | 15.24 | Accepted | 4.16 ± 0.85 | 20.51 | 4.05 ± 0.92 | 22.73 | Accepted |
| 2.1.9 Suffering from genital infections and other sexually transmitted diseases (*Chlamydia* infection) | 4.42 ± 0.67 | 15.25 | 4.26 ± 0.71 | 16.75 | Accepted | 4.41 ± 0.58 | 13.10 | 4.16 ± 0.65 | 15.72 | Accepted |
| 2.1.10 Having many sexual partners or having one partner who is considered high risk (someone with HPV infection or who has many sexual partners) | 4.53 ± 0.60 | 13.16 | 4.42 ± 0.67 | 15.25 | Accepted | 4.58 ± 0.48 | 10.51 | 4.36 ± 0.57 | 13.02 | Accepted |
| **2.2 Basic knowledge of HPV** | 4.47 ± 0.60 | 13.31 | 4.37 ± 0.74 | 16.95 | Accepted | 4.48 ± 0.66 | 14.77 | 4.28 ± 0.7 | 16.32 | Accepted |
| 2.2.1 HPV susceptibility in young women | 4.53 ± 0.68 | 14.98 | 4.42 ± 0.75 | 16.92 | Accepted | 4.42 ± 0.66 | 14.89 | 4.36 ± 0.65 | 14.91 | Accepted |
| 2.2.2 Signs and symptoms of HPV infection | 4.53 ± 0.68 | 14.98 | 4.37 ± 0.81 | 18.51 | Accepted | 4.36 ± 0.65 | 14.91 | 4.00 ± 0.71 | 17.68 | Accepted |
| 2.2.3 Whether HPV infection is treatable | 4.11 ± 0.97 | 23.57 | 3.95 ± 1.10 | 27.84 | Deleted |  |  |  |  |  |
| 2.2.4 Ways to prevent HPV | 4.26 ± 0.71 | 16.75 | 4.11 ± 0.79 | 19.19 | Accepted | 4.36 ± 0.72 | 16.58 | 4.26 ± 0.77 | 17.98 | Accepted |
| 2.2.5 Infection by the HPV is the most important risk factor for cervical cancer | 4.84 ± 0.36 | 7.53 | 4.68 ± 0.65 | 13.94 | Accepted | 4.74 ± 0.43 | 9.07 | 4.68 ± 0.45 | 9.73 | Accepted |
| **2.3 HPV vaccination** | 4.58 ± 0.67 | 14.72 | 4.37 ± 0.67 | 15.24 | Accepted | 4.58 ± 0.58 | 12.59 | 4.21 ± 0.60 | 14.24 | Accepted |
| 2.3.1 HPV vaccine can effectively protect against 70%–90% of cervical cancers | 4.53 ± 0.60 | 13.16 | 4.32 ± 0.73 | 16.90 | Accepted | 4.78 ± 0.40 | 8.36 | 4.71 ± 0.44 | 9.45 | Accepted |
| 2.3.2 Optimal age range for HPV vaccination | 4.32 ± 0.80 | 18.49 | 4.11 ± 0.79 | 19.19 | Accepted | 4.83 ± 0.36 | 7.53 | 4.57 ± 0.58 | 12.68 | Accepted |
| 2.3.3 Women need regular cervical screening even after receiving HPV vaccination | 4.79 ± 0.41 | 8.51 | 4.58 ± 0.67 | 14.72 | Accepted | 4.89 ± 0.30 | 6.18 | 4.83 ± 0.36 | 7.53 | Accepted |
| 2.3.4 Attitudes and intentions toward HPV vaccination | 4.26 ± 0.85 | 19.91 | 4.11 ± 0.72 | 17.48 | Accepted | 4.26 ± 0.70 | 16.34 | 4.05 ± 0.74 | 18.27 | Accepted |
| 1. **Cervical cancer secondary prevention literacy** | 4.75 ± 0.54 | 11.29 | 4.47 ± 0.75 | 16.80 | Accepted | 4.68 ± 0.55 | 11.85 | 4.51 ± 0.49 | 10.91 | Accepted |
| **3.1 Signs and symptoms of cervical cancer** | 4.47 ± 0.60 | 13.31 | 4.26 ± 0.78 | 18.39 | Accepted | 4.21 ± 0.68 | 16.07 | 4.11 ± 0.62 | 15.19 | Accepted |
| 3.1.1 Abnormal vaginal bleeding (when not on your period or after periods have stopped or bleeding after intercourse) | 4.32 ± 0.80 | 18.49 | 4.05 ± 1.05 | 25.91 | Revised^1^ | 4.68 ± 0.45 | 9.73 | 4.56 ± 0.58 | 12.76 | Accepted |
| 3.1.2 Abnormal vaginal discharge (which appears pale, brown, pink, watery, or contains blood) | 4.37 ± 0.87 | 19.94 | 4.26 ± 0.91 | 21.31 | Revised^2^ | 4.52 ± 0.58 | 12.90 | 4.36 ± 0.79 | 18.15 | Accepted |
| 3.1.3 Persistent pelvic pain | 4.11 ± 0.97 | 23.57 | 3.95 ± 1.1 | 27.84 | Deleted |  | | | | |
| **3.2 Cervical cancer screening** | 4.79 ± 0.41 | 8.51 | 4.63 ± 0.58 | 12.55 | Accepted | 4.79 ± 0.40 | 8.31 | 4.68 ± 0.55 | 11.85 | Accepted |
| 3.2.1 Common cervical cancer screening methods | 4.42 ± 0.67 | 15.25 | 4.16 ± 0.81 | 19.53 | Accepted | 4.42 ± 0.66 | 14.89 | 4.05 ± 0.67 | 16.52 | Accepted |
| 3.2.2 Significance of abnormal cervical cancer screening results | 4.21 ± 0.89 | 21.21 | 3.79 ± 0.95 | 25.08 | Accepted | 4.31 ± 0.64 | 14.78 | 4.31 ± 0.74 | 17.20 | Revised^3^ |
| 3.2.3 The most appropriate age to start screening women for cervical cancer | 4.47 ± 0.75 | 16.80 | 4.37 ± 0.74 | 16.95 | Accepted | 4.36 ± 0.72 | 16.58 | 4.15 ± 0.73 | 17.50 | Accepted |
| 3.2.4 The frequency of cervical cancer screening | 4.58 ± 0.67 | 14.72 | 4.47 ± 0.68 | 15.16 | Accepted | 4.38 ± 0.72 | 16.51 | 4.06 ± 0.81 | 19.83 | Accepted |
| 3.2.5 Things you need to know before your first cervical screening | 4.32 ± 0.73 | 16.90 | 4.21 ± 0.83 | 19.76 | Accepted | 4.26 ± 0.62 | 14.55 | 4.01 ± 0.77 | 19.35 | Accepted |
| 3.2.6 Cervical cancer screening experience in the past 3 years | 4.26 ± 0.91 | 21.31 | 4.11 ± 0.85 | 20.75 | Accepted | 4.16 ± 0.57 | 13.75 | 4.05 ± 0.59 | 14.56 | Accepted |
| 3.2.7 Perceived benefits of cervical cancer screening | 4.47 ± 0.82 | 18.30 | 4.21 ± 0.89 | 21.21 | Accepted | 4.51 ± 0.66 | 14.74 | 4.26 ± 0.77 | 17.98 | Accepted |
| 1. **Cervical cancer tertiary prevention literacy** | 4.58 ± 0.49 | 10.78 | 4.42 ± 0.75 | 16.92 | Accepted | 4.53 ± 0.58 | 12.82 | 4.16 ± 0.57 | 13.73 | Accepted |
| **4.1 Seek medical attention on time** | 4.37 ± 0.67 | 15.24 | 4.21 ± 0.52 | 12.37 | Accepted | 4.52 ± 0.58 | 12.90 | 4.36 ± 0.65 | 14.96 | Accepted |
| 4.1.1 Attitude toward timely access to medical care | 4.68 ± 0.73 | 15.57 | 4.53 ± 0.82 | 18.09 | Accepted | 4.58 ± 0.66 | 14.34 | 4.46 ± 0.74 | 16.48 | Accepted |

*Abbreviations:* HIV, human immunodeficiency virus; HPV, human papillomavirus.

1 Early-stage cervical cancer is usually asymptomatic, but as it progresses, irregular vaginal bleeding may occur (either when not on your period, after the period has stopped, or after intercourse).

2 Early-stage cervical cancer is usually asymptomatic, but as it progresses, abnormal vaginal discharge may occur (which appears pale, brown, pink, watery, or contains blood).

3 Next steps after an abnormal cervical cancer screening test.
